# Plasma Levels and Renal Handling of Amino Acids Contribute to Determination of Risk of Mortality or Feed of Ventilation in Patients with COVID-19

**DOI:** 10.3390/metabo12060486

**Published:** 2022-05-27

**Authors:** Gábor Bánfai, Péter Kanizsai, Csaba Csontos, Szilárd Kun, Ágnes Lakatos, Anikó Lajtai, Vanessza Lelovics, Sándor Szukits, Péter Bogner, Attila Miseta, István Wittmann, Gergő A. Molnár

**Affiliations:** 1 Department of Emergency Medicine, University of Pécs Medical School, 7624 Pécs, Hungary; banfai.gabor@pte.hu (G.B.); kanizsai.peter@pte.hu (P.K.); 2 Department of Anesthesiology and Intensive Care, University of Pécs Medical School, 7624 Pécs, Hungary; csontos.csaba@pte.hu; 3 2nd Department of Medicine and Nephrology-Diabetes Center, University of Pécs Medical School, 7624 Pécs, Hungary; kun.szilard@pte.hu (S.K.); molnar.gergo@pte.hu (G.A.M.); 4 Department of Laboratory Medicine, University of Pécs Medical School, 7624 Pécs, Hungary; lakatos.agnes@pte.hu (Á.L.); lajtai.aniko@pte.hu (A.L.); lelovics.vanessza@pte.hu (V.L.); miseta.attila@pte.hu (A.M.); 5 Department of Medical Imaging, University of Pécs Medical School, 7624 Pécs, Hungary; szukits.sandor@pte.hu (S.S.); bogner.peter@pte.hu (P.B.)

**Keywords:** COVID-19, predictor, in-hospital mortality, need of mechanical ventilation, amino acid plasma levels, fractional excretion

## Abstract

COVID-19 infection may lead to serious complications, e.g., need for mechanical ventilation or death in some cases. A retrospective analysis of patients referred to our COVID Emergency Department, indiscriminately, was performed. A routine lab analysis measured amino acids in plasma and urine of patients. Data of surviving and deceased patients and those requiring or not requiring mechanical ventilation were compared, and logistic regression analyses have been performed. Deceased patients were older, had higher blood glucose, potassium, AST, LDH, troponin, d-dimer, hsCRP, procalcitonin, interleukin-6 levels (*p* < 0.05 for all). They had lower plasma serine, glycine, threonine, tryptophan levels (*p* < 0.01), higher tyrosine and phenylalanine levels (*p* < 0.05), and higher fractional excretion of arginine, methionine, and proline (*p* < 0.05) than survivors. In a regression model, age, severity score of COVID-pneumonia, plasma levels of threonine and phenylalanine were predictors of in-hospital mortality. There was a difference in ventilated vs. non-ventilated patients in CT-scores, glucose, and renal function (*p* < 0.001). Using logistic regression, CT-score, troponin, plasma level, and fractional excretion of glycine were predictors of ventilation. Plasma levels and renal excretion of certain amino acids are associated with the outcome of COVID-19 infection beside other parameters such as the CT-score or age.

## 1. Introduction

The infection caused by the novel coronavirus SARS-CoV-2 has become a pandemic in the last year, providing an immense burden on the healthcare system and economics worldwide [1,2]. Emergency departments (ED) and intensive care unit (ICU) facilities are particularly affected [3]. The infection may occur in various forms, from banal upper airway infection with or without the loss of smell or taste to bilateral, extensive pneumonia requiring mechanical ventilation, in some cases with gastrointestinal symptoms, and may eventually even lead to death of the patients. Beyond the airway-related symptoms, affection of other organ systems is also frequent, as patients with COVID-19 frequently have complications related to the cardiovascular, cerebrovascular, and neurologic systems, and the disease may also lead to arterial or venous thromboembolism [4]. Furthermore, long-term consequences of COVID-19 have increasingly surfaced [5].

Since the outcome of the infection may range from spontaneous healing to death, it would be very important to provide tools to estimate the severity of the disease (e.g., risk of mortality or the need of mechanical ventilation) in patients with COVID-19. There have been attempts to do this [4,6,7,8,9,10,11,12,13,14,15,16,17,18,19], but the studies aiming to find predictors of outcomes have shown controversial data.

Among affected organs, besides the lungs, metabolic factors and the renal function may also be affected by SARS-CoV-2 according to studies [20,21,22]. Additionally, a new terminology has been proposed, i.e., coronavirus-associated nephropathy (COVAN) [23]. Such patients may show a decline in GFR, hematuria, and proteinuria as well. The involvement of the proximal tubuli in the kidneys is especially highlighted by the literature. Data suggest that renal involvement due to the viral infection may determine outcomes and risk of kidney injury [22,24]. 

Beyond COVID-19, an acute kidney damage involving the proximal tubuli may be characterized by several derangements such as metabolic acidosis, electrolyte imbalances, renal glucosuria, or aminoaciduria [25,26]. Detection of aminoaciduria is currently not a routinely used diagnostic tool, as it requires analysis of the levels of amino acids in the plasma and from the urine. By determining the creatinine levels in the serum and urine, and cross-dividing the above-mentioned parameters, fractional excretion of the individual amino acids can be determined [26]. 

In our study, we aimed to determine predictors of mortality and the need for ventilation in patients referring to an ED using not only classical clinical and laboratory parameters such as the score on computed tomography of the lungs and inflammatory parameters, but also plasma levels and fractional excretion of amino acids.

## 2. Results

### 2.1. General Study Population

A total of 191 patients were covered in our study. Among them, 26 (13.6%) died and 15 (7.9%) patients needed mechanical ventilation. Out of the mechanical ventilation group, 13 (86.7%) died and reversely, 50.0% of the non-survival patients needed mechanical ventilation. Accordingly, not all deceased patients (*n* = 26) were mechanically ventilated (*n* = 15); there were other causes of death in 11 cases. All 11 cases had an age of 79–90 years, were in a bad general condition already at admittance. Additionally, some patients, based on their age, known severe comorbidities, and general condition, had a very low life expectancy, and thus they were not candidates for treatment at the ICU. These 11 patients died of predominantly non-pulmonary causes, as most of these patients had a combination of multiple severe diseases such as hypotension, sepsis, requirement of vasopressor agents, renal failure, fever, dehydration, consequences of immobility, and nosocomial infections.

Those baseline characteristics in which the groups did not differ according to survival and mechanical ventilation requirements are shown at in Supplementary Tables S1 and S3, respectively. Parameters with an area under receiver-operating curve (ROC curve) value higher than 0.700 that were enrolled in logistic regression models, are shown in Table 1 (survival) and Table 3 (need for mechanical ventilation), respectively. Serum levels and fractional excretions of amino acids were also correlated with several important clinical parameters, such as age, renal function, markers of inflammation or cell injury. These data are presented in Supplementary Tables S5 and S6. Some parameters showed a positive, others a negative correlation with inflammation. Fractional excretion of many parameters showed a positive correlation with markers of renal function, however for several other amino acids, the opposite was true.

### 2.2. In-Hospital Mortality

Clinical parameters that differed according to mortality were age, computed tomography severity index (CTSI), serum potassium, plasma glucose, blood urea nitrogen (BUN), serum creatinine, aspartate aminotransferase (AST), troponin T, D-dimer, lactate dehydrogenase (LDH), high sensitivity C-reactive protein (hsCRP), procalcitonin, interleukin-6 (IL-6), international normalized ratio (INR), white blood cell count (WBC), neutrophil %, neutrophil count, red blood cell distribution width (RDW), and neu/mo. Those showing positive association with mortality were hypertension, diabetes mellitus (DM), cardiovascular (CV) disease, malignancy, and need for mechanical ventilation. Among amino acid parameters, values of plasma Asp, Tyr, Phe, and fractional excretion (FE) of Asn, Arg, Met, Ile, Leu, Lys, and Pro were higher in non-survivor patients (Table 1, Supplementary Table S1).

Clinical parameters with lower values in non-survivals were eGFR, lymphocyte %, lymphocyte count, ly/neu, monocyte %, basophil %, Hb and MCHC. Among amino acid parameters, plasma Ser, Gly, Thr, and Trp levels and plasma Tyr/Phe ratio were lower in non-survivor patients (Table 1, Supplementary Table S1).

**Figure 1:**
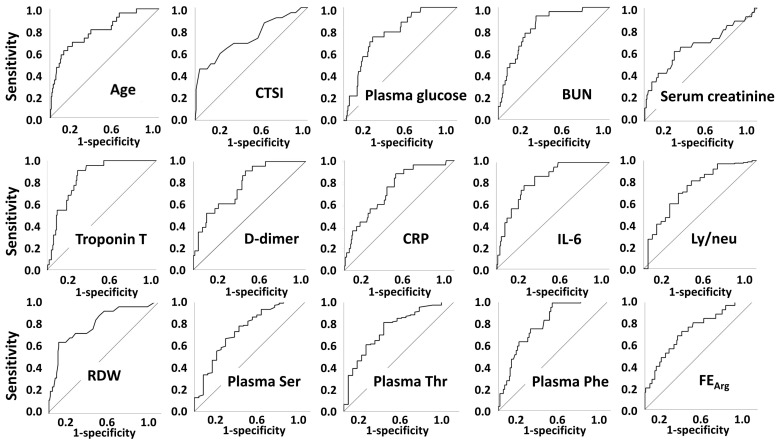
ROC curves of parameters with area under the ROC curve above 0.700 for in-hospital mortality. Abbreviations: CTSI, CT severity index; BUN, blood urea nitrogen; eGFR, estimated glomerular filtration rate; hsCRP, high-sensitivity C-reactive protein; IL-6, interleukin 6; RDW, red blood cell distribution width; Ly/neu, lymphocyte/neutrophil; FE, fractional excretion.

Parameters with significant inter-group differences were further tested using ROC curve analyses. Among them, age, CTSI, plasma glucose, BUN, eGFR, troponin T, D-dimer, hsCRP, procalcitonin, IL-6, neutrophil %, neutrophil count, lymphocyte %, RDW, ly/neu, plasma Ser, plasma Thr, and FE of Arg had an area under the ROC curve above 0.700 (Figure 1, Supplementary Table S2).

The strongest parameters were enrolled to logistic regression models, separately with serum creatinine or BUN. 

Model 1 included age, serum creatinine, CTSI, plasma glucose, troponin T, D-dimer, hsCRP, IL-6, RDW, Ly/neu ratio, plasma Ser levels, plasma Thr levels, plasma Phe levels, and fractional excretion of Arg.

Model 2 included age, BUN, CTSI, plasma glucose, troponin T, D-dimer, hsCRP, IL-6, RDW, Ly/neu ratio, plasma Ser levels, plasma Thr levels, plasma Phe levels, and fractional excretion of Arg.

In both models, age, CTSI, plasma Thr, and plasma Phe levels were independent predictors of in-hospital mortality (Table 2).

From the regression coefficients, the probability of events could be calculated using the equation of logistic regression. The resulting calculations were incorporated into a simple Excel spreadsheet-based calculator that can be found as an on-line supplement (Supplementary Figure S1).

### 2.3. Mechanical Ventilation

Clinical parameters that differed according to the need for mechanical ventilation were CTSI, plasma glucose, BUN, serum creatinine, AST, troponin T, D-dimer, LDH, hsCRP, procalcitonin, ferritin, IL-6, INR, WBC, neutrophil %, neutrophil count, RDW, and neu/mo. Among amino acid parameters, plasma Asp, Glu, Phe, FE of Arg, Met, Leu, Lys, and Pro were higher in mechanically ventilated patients (Table 3, Supplementary Table S3).

Clinical parameters with lower levels in patients needing mechanical ventilation were eGFR, lymphocyte %, lymphocyte count, ly/neu, monocyte %, and basophil %. Among amino acid parameters, plasma Ser, Gln, Hys, Gly, Thr, Trp levels, plasma Tyr/Phe ratio, urinary total AA/creatinine, FE of Gln, His, and Gly were lower in mechanically ventilated patients (Table 3, Supplementary Table S3).

**Figure 2:**
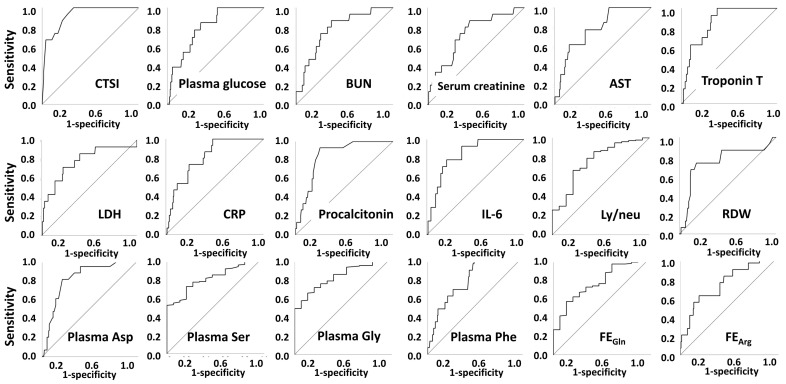
ROC curves of parameters with area under the ROC curve above 0.700 for mechanical ventilation. Abbreviations: CTSI, CT severity index; BUN, blood urea nitrogen; eGFR, estimated glomerular filtration rate; AST, aspartate transaminase; LDH, lactate dehydrogenase; hsCRP, high-sensitivity C-reactive protein; IL-6, interleukin 6; WBC, white blood cell; RDW, red blood cell distribution width; Ly/neu, lymphocyte/neutrophil; Neu/mo, neutrophil/monocyte; FE, fractional excretion.

Parameters with significant between-group differences were further tested using ROC curve analyses. Among them, CTSI, plasma glucose, BUN, serum creatinine, eGFR, AST, troponin T, LDH, hsCRP, procalcitonin, IL-6, WBC, neutrophil %, neutrophil count, lymphocyte %, monocyte %, basophil %, RDW, ly/neu, neu/mo, plasma Asp, Ser, Gln, His, Gly, Thr, Trp, Phe levels, urinary total AA/creatinine, FE of Gln, Gly, Arg, and Pro had an area under the ROC curve above 0.700 (Figure 2, Supplementary Table S4).

The strongest parameters were enrolled to logistic regression models, separately with serum creatinine or BUN, in both cases with or without LDH.

As for the need of mechanical ventilation, Model 1 included serum creatinine, CTSI, plasma glucose, troponin T, hsCRP, IL-6, Ly/neu, plasma Ser, plasma Gly, plasma Phe, FE Gly, and FE Arg.

Model 2 included BUN, CTSI, plasma glucose, troponin T, hsCRP, IL-6, Ly/neu, plasma Ser, plasma Gly, plasma Phe, FE Gly, and FE Arg.

Model 3 included serum creatinine, CTSI, plasma glucose, troponin T, LDH, hsCRP, IL-6, Ly/neu, plasma Ser, plasma Gly, plasma Phe, FE Gly, and FE Arg.

Model 4 included BUN, CTSI, plasma glucose, troponin T, LDH, hsCRP, IL-6, Ly/neu, plasma Ser, plasma Gly, plasma Phe, FE Gly, and FE Arg.

In models without LDH, the CTSI and plasma Gly were found to be independent predictors of mechanical ventilation, while in the model including serum creatinine and LDH, none of the included parameters were significant predictors; however, the model itself was significant. 

In a model with BUN and LDH, CTSI, troponin T, plasma Gly, and FE of Gly were independent predictors of mechanical ventilation (Table 4).

With the same approach as in case of mortality, as well as in case of need of ventilation, the resulting equations were incorporated into the same Excel-based calculator (Supplementary Figure S1).

## 3. Discussion

In our retrospective analysis of data of COVID-19 patients, we were looking for predictors of in-hospital mortality and the need for mechanical ventilation. We found that besides the classical demographic and laboratory parameters, values related to plasma levels or renal handling of amino acids also contributed to risk determination.

Regarding co-morbidities, hypertension, diabetes mellitus, CV disease, malignancies, but not kidney disease or autoimmune diseases, were associated with mortality. 

Data on the prognosis of COVID-19 are quite heterogenous, similar to the studies the data originate from. Data of the literature are also quite heterogenous in terms of predictors of severity of mortality of COVID-19. This has to do with the geographical differences, the healthcare systems, the time of analysis, the currently dominating strain of SARS-CoV2, etc. For example, we know that in the first and second waves of COVID-19, elderly patients were rather infected and hospitalized, while with emerging variants in the third and fourth wave, also markedly younger patients were hospitalized or even died of the disease, hence the large variance. At the time of sample collection to our study, variant B.1.1.7 (the alpha variant) dominated among the infected patients [27], thus our patients were homogenous in that regard. Additionally, by the time the data were collected, the roll-out of the vaccines was just initiated and approx. 0.1% of the total population was vaccinated [27]; in fact, none of our affected patients were vaccinated yet. Therefore, vaccination status could not interfere with our results.

Some studies and reviews analyzed the predictive value of only one or two parameters, and their usefulness in predicting outcomes of COVID-19, e.g., the role of platelet/lymphocyte ratio [28], HbA_1c_ [29], vitamin D3 levels [30], obesity [31], d-dimer [32], lymphocyte, or neutrophil count [33]. Other studies tried to provide predictors from a more complete set of clinical markers.

Our study is totally unique in the regard that it uses amino acid parameters both from sera and urine of patients with COVID-19. Additionally, according to our latest search of the literature, no other study has used amino acid parameters in COVID-19 with these endpoints (mortality and need of mechanical ventilation). 

Of note, one study has been published recently, investigating 112 patients with COVID-19 infection, where serum levels of Leu, Ile, taurine, trans-4-hydroxy-Pro, Pro, Thr, Gln, His, ornithine, and citrulline were significantly lower, while levels of Phe and L-2-aminobutyric acid were significantly higher in patients with severe infection as compared to non-COVID controls [34]. As compared to that, we found that plasma levels of Ser and Thr were lower and Phe plasma levels were higher in the deceased vs. survivor patients. In our study, the levels of other amino acids did not differ significantly between survivors and non-survivors. As for the need of ventilation, only plasma levels of Ser and Gly were lower, while levels of Asp and Phe were higher in patients requiring mechanical ventilation as compared to those not requiring ventilation. Thus, our data on Thr and Phe are in line with the cited reference. We must emphasize that this study did not investigate urinary levels of the amino acids and also did not take into account other classical clinical predictors such as age, inflammatory parameters, CTSI, etc. 

Another study analyzed COVID-19 cases (n = 32) in a longitudinal manner, comparing serum amino acid levels in the acute phase of COVID-19 vs. the recovery phase of the disease [35]. They found lower levels of 4-hydroxy-Pro, citrulline, and Gln in the active phase, while higher levels of Glu, Phe, Pro, and taurine were found in the active phase vs. the recovery phase. They do not provide data on Asp or Gly levels, two parameters that we found to differ between ventilated vs non-ventilated cases. The Phe data are fully in line with our dataset and results.

In one publication, the value of CTSI above 18 was associated with mortality [14]. In our analysis, the cut-off value for mortality was 13.5, while the CTSI cut-off value for need of ventilation was 11.5. In the multivariate analysis containing amino acid parameters, the CTSI remained in the final model concerning mortality. For need of ventilation, the CTSI had the highest discriminating power (ROC AUC 0.928), as expected, and remained an independent predictor in the logistic regression.

A study from the early COVID-19 era from Wuhan found that d-dimer was associated with mortality. The cut-off was 2000 μg/L, and the hazard ratio was 51.5 [36]. In our study, when considering d-dimer alone, it was associated with mortality, with a cut-off of 751 μg/L; however, in the multivariate analyses containing amino acid parameters, d-dimer dropped out. As for need of ventilation, the discriminating ability of d-dimer was low (AUC < 0.70), thus it was not included in the multivariate regression.

In a further analysis, cardiac involvement and as a marker, high troponin levels predicted mortality. It is believed that beyond respiratory failure, cardiovascular insults may be the most important causes of death for patients with COVID-19. We found that troponin-T values were associated with high mortality with a good predictive value (ROC AUC 0.850), and the same was true for need of ventilation (ROC AUC 0.851). However, troponin values dropped out in the multivariate analysis, including the amino acid parameters.

Hematological parameters, such as neutrophilia, low lymphocyte count or high neutrophil/lymphocyte (neu/ly) ratio seem to be associated with worse outcomes of COVID-19. The latter was investigated in a research paper, and a more than 15 times increased risk was found for the highest neu/ly ratio [19]. Another study found that anemia and thrombocytopenia were associated with poor prognosis [37]. We found that the ly/neu ratio was associated with worse mortality and higher need of ventilation; however, this parameter either dropped out or was not significant in the multivariate analysis. Additionally, the neutrophil/monocyte ratio was suggested as a good predictor [38], but we found a worse discriminating power than that of the neu/ly ratio, therefore it was not used in further analysis.

Further studies analyzed several demographic and clinical parameters at the same time and tried to find predictors of mortality. In most studies, age was an important discriminating factor. In some analyses, cut-off values have been provided, such as >55 years [15], >60 years [39], >65 years [11,40], >70 years [41], >75 years [42], and >78 years [7]. Concerning our patients, age >72 years was a risk factor of mortality, while it was not a risk factor for the need of ventilation. As for mortality, however, age remained an independent predictor in the multivariate analysis.

Carbohydrate disorders and higher glucose values are also suspected determinants of COVID-19 outcomes [9,41,42,43,44]. We found that the presence of diabetes mellitus was associated with both mortality and need for ventilation. A random plasma glucose of 7.60 and 7.01 discriminated for the two outcomes, respectively. 

From the inflammatory parameters, hsCRP is probably the most widely studied in the case of COVID-19, and it is universally accepted as a good predictor [6,12,16,43,44,45,46,47]. We found that a value of 42 mg/L is the optimal cut-off for mortality and ventilation as well. However, in the multivariate analysis, it dropped out.

Another inflammatory parameter, IL-6, also proves to be a good marker for estimating the severity of COVID-19, as verified in a review and a meta-analytic study [18,43]. In our analysis, it served one of the highest discriminating factors for mortality (ROC AUC: 0.861) and for the need of ventilation (ROC AUC: 0.865). However, it turned out not to be significant in regression models.

The kidneys also seem to be involved in COVID-19. In silico as well as post-mortem analyses found that not only the binding proteins of SARS-CoV-2 (such as angiotensin converting enzyme 2—ACE2-, cathepsin L, or transmembrane serine protease 2), but also the genome of the virus could be detected in the kidneys [20,22]. Further analysis verified the co-localization of SARS-CoV-2 nucleocapsid protein and ACE2. Moreover, in autopsy reports of patients deceased of COVID-19, severe acute tubular necrosis has been found, but also peritubular capillaries seemed to be involved [21]. The latter could also be provoked by inflammation and endothelial injury. Furthermore, collapsing glomerulopathy has been described in cases with COVID-19 infection [48,49]. In terms of the severity of COVID-19, renal function was a frequently found predictor of outcomes [6,11,15,18,42,43,50,51]. More specifically, markers of proximal tubular damage such as glucosuria and light proteinuria were also found to be associated with the severity of COVID-19 [52,53]. We used markers of tubular handling as markers of kidney injury and found that renal handling of glycine and arginine were related to the need for ventilation. More severe systemic inflammation could lead to multi-organ failure contributing to acute kidney injury [24,49], but may, at the same time, be associated with mortality or need of ventilation.

Our data do not provide a causal relationship between the amino acid parameters and clinical markers; these are mainly associations. Additionally, it cannot be ruled out whether changes in the amino acid parameters influenced the clinical markers, or rather vice versa, clinical parameters such as inflammation or renal function influenced the amino acid parameters.

As judged by the correlation analysis, the amino acids behave differently, e.g., plasma levels of several amino acids (such as Asn, Ser, Gln, His, Gly, Thr) showed a negative correlation with troponin, d-dimer, or CRP and may represent a catabolic state or increased metabolism of these amino acids; others (such as Asp or Phe) did the opposite; they had a positive correlation with the same parameters, and rather seem proinflammatory parameters. 

Concerning levels of amino acids in the blood, data of the literature point to a generalized inflammation and sepsis, where a smaller proportion of amino acids (such as Arg, Asp, Gln, Glu, Phe) showed an increasing tendency as compared to controls, while the larger proportion of amino acids (such as Asn, His, Ile, Leu, Lys, Pro, Thr, Trp, Tyr, Val) was in fact lower in patients with generalized inflammation/sepsis as compared to controls. When comparing sepsis survivors vs. non-survivors, levels of Phe were higher, whereas levels of Arg, Glu, Ser, and Trp were lower in the deceased patients. Furthermore, sulphur-containing amino acids (Cys, Tau, Met) were lower in the non-surviving patients [54]. 

Recent evidence on the role of amino acids in inflammatory processes in mammals was summarized, and data suggest that Thr is needed for the synthesis of mucin and antibodies [55]. In our study, survivors had higher levels of serum Thr than the non-survivors. Additionally, in the multivariate model, Thr level in the blood showed a negative association with mortality.

Arg is a well-known substrate for the nitric oxide synthase (NOS) enzyme, and the inducible form, iNOS, plays an important role in the defensive role of immune cells [55,56]. We found a markedly higher FE of Arg in the deceased patients and in those requiring mechanical ventilation. This may be a sign of renal loss of Arg, which may have a negative impact of immune defense. 

Gly plays a role in the regulation of proliferation and cytokine synthesis of leukocytes, among others, via a glycine-gated chloride channel [56]. Concerning our own data, serum levels of Gly were lower despite a lower FE of Gly in those requiring mechanical ventilation. A study compared sepsis to SIRS, and higher levels of Asp and Glu, while lower levels of Val, Ser, and Leu were found in sepsis [57]. 

Phe is also known to be a co-factor promoting the synthesis of tetrahydrobiopterin and thus this may lead to a higher rate of iNOS activity and a more active inflammatory process. In our data, we found higher plasma Phe levels, but lower Gly, Ser, Tre, and Trp levels in the non-survivors of COVID-19. Similarly, higher levels of Phe, Asp, and Glu, and lower levels of Ser, Gln, His, Gly, Thr, and Trp were found in patients needing ventilation.

Gly and Pro may also be of interest since the former makes up to one-third and the latter makes up approx. 20% (together > 50%) of all amino acid residues in collagen chains of the lungs. Synthesis of these amino acids is also coupled to levels of other amino acids such as Gln, Glu, Arg, and Ser [58]. Furthermore, proline-glycine-proline peptide fragments of collagen may modulate inflammation in acute lung injury [59]. 

Data of the literature suggest that supplementation with selected amino acids or a mixture of them may have an impact on inflammatory processes in humans as well [56]. More specifically, data on supplementation of COVID-19 patients with micronutrients or amino acids (including Gln) have been presented in a review article [60], according to which two studies failed to demonstrate benefit of supplementation with Gln in patients with COVID-19.

Risk factors of COVID-19 infections may be different in different populations with different levels of development. Indeed, data of the literature suggest that level of development and country income rate may be strongly related to COVID-19 mortality [61]. Thus, our study results including the risk calculator developed by our workgroup need to be validated in countries with higher and lower incomes as well.

## 4. Patients and Methods

### 4.1. Patients and Parameters

Our study is an investigator-initiated, single-site, retrospective database-analyzing clinical study. Patients with proven SARS-CoV-2 infection, visiting the Emergency Care Unit of the Coronavirus Care Centre of the Clinical Centre University of Pécs between 1 Jan 2021 and 30 Apr 2021 were involved without any further selection. The study protocol has been approved by the Research Ethics Committee of the Clinical Centre, University of Pécs (application No. 8744—PTE2021). Data generated exclusively during routine patient care were recorded, retrospectively.

Basic demographic parameters (age, gender), comorbidities (hypertension, diabetes mellitus (DM), cardiovascular (CV) disease, kidney disease, malignancy, autoimmune disease) and data on COVID-19 disease associated outcomes (in-hospital total mortality and need for mechanical ventilation) were obtained. Beyond these, the following clinical parameters were recorded: Computed Tomography Severity Index for COVID-19 associated pneumonia (CTSI), serum sodium (Na), serum potassium (K), plasma glucose, blood urea nitrogen (BUN), serum creatinine, urinary creatinine, estimated glomerular filtration rate (eGFR), serum aspartate transaminase (AST), serum alanine transaminase (ALT), serum troponin T, D-dimer, serum lactic dehydrogenase (LDH), serum high-sensitivity C-reactive protein (hsCRP), serum procalcitonin (PCT), serum ferritin, serum interleukin 6 (IL-6), international normalized ratio (INR), white blood cell (WBC), neutrophil %, neutrophil count, lymphocyte %, lymphocyte count, lymphocyte/neutrophil (ly/neu), monocyte %, monocyte count, neutrophil/monocyte (neu/mo), eosinophil %, eosinophil count, basophil %, basophil count, red blood cell (RBC), hemoglobin (Hb), hematocrit (Htc), mean corpuscular volume (MCV), mean corpuscular hemoglobin (MCH), MCHC (mean corpuscular hemoglobin concentration), red blood cell distribution width (RDW), thrombocyte, mean platelet volume (MPV), plasma and urinary levels and fractional excretions (FE) of amino acids: aspartic acid (Asp), glutamic acid (Glu), asparagine (Asn), serine (Ser), glutamine (Gln), histidine (Hys), glycine (Gly), threonine (Thr), arginine (Arg), alanine (Ala), tyrosine (Tyr), cysteine (Cys), valine (Val), methionine (Met), tryptophane (Trp), phenylalanine (Phe), isoleucine (Ile), leucine (Leu), lysine (Lys), proline (Pro). The measurement of amino acid levels was carried out as a routine laboratory measurement provided by the Department of Laboratory Medicine for diseases with a higher chance of renal involvement such as COVID-19 infection, acute kidney injury, sepsis, or multi-organ failure. All patients visiting the Emergency Care Unit (ECU) of the Coronavirus Care Centre of the Clinical Centre University of Pécs proven to be COVID-positive could be tested for amino acid levels from plasma and urine samples.

The aim of the study was to find whether we could predict from the parameters determined when entering the COVID-ECU which patients would require mechanical ventilation or would die during the hospitalization. Therefore, clinical data of the patients were collected at the entry to the Emergency Department, regardless of the onset of the symptoms.

When calculating fractional excretion of a substance, its clearance is divided by that of creatinine, and 24 h urine output eventually drops out of the equation, thus only serum levels and urine concentrations of the substances are needed. Since determination of urinary amino acid concentrations were performed using spot urine and not a 24 h-collected urine, the calculated fractional excretion values are rather estimates of the true FE values.

### 4.2. Amino Acid Analysis from Plasma and Urine Samples

We have measured amino acids from EDTA plasma and urine with an Ultra-High Performance/Pressure Liquid Chromatography (UPLC) method after derivatization followed by fluorescence detection according to a previously well-described and accepted method [62]. The sample preparation was a simple deproteinization with acetonitrile (addition of 300 µL acetonitrile to a sample of 200 µL plasma or urine, followed by centrifugation 8360 rpm for 4 min) The derivatization for the primer amino acids was the standard OPA (ortho-phtalaldehyde) and MPA (mercapto propionic acid) method, for Pro the FMOC (9-Fluorenylmethoxycarbonyl chloride) method. We used a Shimadzu Nexera UPLC (LC30) system (Shimadzu Manufacturing Inc., Canby, OR, USA) with Kinetex^®^ 2.6µm EVO C18 100A 150 × 4.6 mm column (Phenomenex Inc., Torrance, CA, USA). The eluent was a gradient of phosphate puffer pH: 6.2 (0.02 mol/L) and acetonitrile: methanol: water (40:45:15) The fluorescence detection was on an excitation wavelength of 350 nm, and an emission wavelength of 450 nm and excitation wavelength of 266 nm, and emission wavelength of 305 nm, respectively, with a RF-20AXS detector.

### 4.3. Statistical Analysis

Two main clinical outcomes served as grouping parameters: survival and mechanical ventilation state. The following groups were established and compared: survivors vs. non-survivors, and no mechanical ventilation vs. need for mechanical ventilation. Clinical parameters of parallel groups were compared using Mann–Whitney U test as they had been found to have non-normal distribution, according to results of Kolmogorov–Smirnov test. Parameters with significant between-group differences in the U test were analyzed using receiver operating characteristic curve (ROC) statistic. In the logistic regression models, those parameters were examined, which had an area under ROC curve > 0.700. Logistic regressions were performed using backward likelihood ratio method as suggested by the literature [63,64]. 

Based on the results of logistic regressions, we aimed to elaborate a score system involving various clinical parameters, aiming to estimate expected clinical outcome. We used the solution of the logistic regression equation to estimate probability of the event (in-hospital mortality or need for ventilation), i.e., $p=\frac{e^{z}}{1+e^{z}}=\frac{e^{\alpha +\beta_{1}*x_{1}+\beta_{2}*x_{2}\ldots {\;\beta }_{n}*x_{n}}}{1+e^{\alpha +\beta_{1}*x_{1}+\beta_{2}*x_{2}\ldots {\;\beta }_{n}*x_{n}}}$, where *p* is the probability of the event, *z* is the logit of the odds, *α* is the constant in the regression equation, *β_1-n_* are the regression coefficients of the predictors *X_1−n_* [65].

A ‘*p*’ value < 0.05 was considered as significant. Analyses were performed using IBM SPSS Statistics package 26 (Berkeley, CA, USA).

## 5. Conclusions

To conclude, serum levels and renal handling of amino acids could contribute to risk determination of COVID-19 beyond classical markers such as age, signs of inflammation, or severity of lung involvement.

## Figures and Tables

**Table 1:** Characteristics of study population by survival state. Only parameters with a significant difference between the groups are shown here. All other parameters are listed in Supplementary Table S1.

		Survivors	Non-Survivors	
Parameter	Unit	Median (q1–q3)	Median (q1–q3)	*p*
Number of patients	n	163	26	
Age	year	58.5 (45.4–67.2)	76.2 (63.9–83.5)	<0.001
Diabetes (yes/no)	n/n (%/%)	35/130 (21.2/78.8)	11/15 (42.3/57.7)	0.019
CV disease (yes/no)	n/n (%/%)	28/137 (17.0/83.0)	10/16 (38.5/61.5)	0.011
Hypertension (yes/no)	n/n (%/%)	79/86 (47.9/52.1)	60/6 (76.9/23.1)	0.006
Cancer (yes/no)	n/n (%/%)	8/157 (4.8/95.2)	5/21 (19.2/80.8)	0.019
Renal replacement therapy	n [%]	1 [0.6]	3 [11.5]	0.008
Mechanical ventilation	n [%]	2 [1.2]	13 [50.0]	<0.001
CTSI		9 (6–13)	16 (8–21)	0.001
Plasma glucose	mmol/L	6.34 (5.60–7.80)	8.91 (7.30–9.80)	<0.001
BUN	mmol/L	4.4 (3.4–6.1)	9.3 (6.4–15.9)	<0.001
Serum creatinine	µmol/L	80 (68–95)	97 (74–169)	0.008
Troponin T	µg/L	6.8 (4.0–12.3)	25.8 (13.6–38.5)	<0.001
D-dimer	µg/L	680 (411–1118)	1697 (832–4316)	<0.001
hsCRP	mg/L	36.3 (9.9–98.7)	109.5 (61.5–180.7)	<0.001
IL-6	pg/mL	21.8 (9.8–48.4)	96.9 (53.5–225.7)	<0.001
RDW	%CV	12.8 (12.2–13.5)	14.6 (13.1–15.1)	<0.001
Ly/neu		0.263 (0.163–0.427)	0.130 (0.078–0.214)	<0.001
Plasma Ser	µmol/L	67.0 (59.6–76.4)	56.6 (47.9–65.0)	<0.001
Plasma Thr	µmol/L	75.7 (65.7–91.0)	61.4 (50.0–72.0)	<0.001
Plasma Phe	µmol/L	77.0 (65.0–96.0)	103.6 (84.0–123.8)	<0.001
FE Arg	%	3.069 (1.750–5.120)	5.614 (3.487–10.112)	<0.001

*p*, survivors vs. non-survivors, using Mann–Whitney U test in case of continuous and chi-square or Fisher’s exact test in case of categorical variables. Abbreviations: CTSI, CT severity index; BUN, blood urea nitrogen; hsCRP, high-sensitivity C-reactive protein; IL-6, interleukin 6; RDW, red blood cell distribution width; Ly/neu, lymphocyte/neutrophil; FE, fractional excretion.

**Table 2:** Predictors of mortality.

Parameter	B	*p*
**Model 1**		
Age	0.148	<0.001
CTSI	0.294	<0.001
Plasma Thr	−0.077	0.009
Plasma Phe	0.036	0.004
**Model 2**		
Age	0.148	<0.001
CTSI	0.296	<0.001
Plasma Thr	−0.077	0.009
Plasma Phe	0.036	0.004

Logistic regression using backward LR method; B denotes the regression coefficient, while *p* denotes the statistical significance of the individual predictors. Model 1: age, serum creatinine, CTSI, plasma glucose, troponin T, D-dimer, hsCRP, IL-6, RDW, Ly/neu, plasma Ser, plasma Thr, plasma Phe, FE Arg. Model 2: age, BUN, CTSI, plasma glucose, troponin T, D-dimer, hsCRP, IL-6, RDW, Ly/neu, plasma Ser, plasma Thr, plasma Phe, FE Arg. Abbreviations: CTSI, CT severity index; BUN, blood urea nitrogen; hsCRP, high-sensitivity C-reactive protein; IL-6, interleukin 6; RDW, red blood cell distribution width; Ly/neu, lymphocyte/neutrophil; FE, fractional excretion.

**Table 3:** Characteristics of study population by mechanical ventilation state. Only parameters with a significant difference between the groups are shown here. All other parameters are listed in Supplementary Table S3.

		No Mechanical Ventilation	Mechanical Ventilation	
Parameter	Unit	Median (q1–q3)	Median (q1–q3)	*p*
Number of patients	n	176	15	
Diabetes (yes/no)	n/n (%/%)	38/138 (21.6/78.4)	8/7 (53.3/46.7)	0.006
Hypertension (yes/no)	n/n (%/%)	87/89 (49.4/50.6)	12/3 (80.0/20.0)	0.030
CTSI		9 (6–13)	20 (16–22)	<0.001
Plasma glucose	mmol/L	6.38 (5.69–8.44)	9.05 (7.72–11.85)	<0.001
BUN	mmol/L	4.53 (3.49–6.6)	7.58 (6.22–13.43)	<0.001
Serum creatinine	µmol/L	80 (68–98)	96 (87–156)	0.004
Troponin T	µg/L	7.23 (4.06–14.68)	26.58 (13.56–42.74)	<0.001
LDH	U/L	535 (409–714)	834 (631–1433)	<0.000
hsCRP	mg/L	38.7 (10.0–99.5)	155.6 (82.2–192.3)	<0.001
Procalcitonin	ng/mL	0.06 (0.04–0.11)	0.17 (0.13–0.47)	<0.001
IL-6	pg/mL	24.0 (10.2–56.6)	109.2 (70.4–233.9)	<0.001
RDW	%CV	12.9 (12.3–13.6)	14.6 (13.6–14.9)	0.001
Ly/neu		0.254 (0.149–0.424)	0.114 (0.079–0.243)	0.008
Plasma Asp	µmol/L	5.0 (4.0–6.2)	8.0 (7.0–9.3)	<0.001
Plasma Ser	µmol/L	66.97 (58.9–76.2)	55.2 (49.5–60.9)	<0.001
Plasma Gly	µmol/L	117.2 (97.9–142.2)	93.0 (83.1–100.5)	<0.001
Plasma Phe	µmol/L	79.0 (66.3–98.0)	103.2 (84.0–126.4)	<0.001
Urinary total AA/creatinine	µmol/mmol	0.252 (0.187–0.349)	0.184 (0.142–0.204)	<0.001
FE Gln	%	0.550 (0.372–0.742)	0.386 (0.248–0.460)	0.002
FE Gly	%	2.586 (1.819–3.980)	1.763 (1.350–2.000)	0.002
FE Arg	%	3.177 (1.776–5.216)	7.163 (3.594–9.072)	0.003

*p*, no mechanical ventilation vs. mechanical ventilation, using Mann–Whitney U test in case of continuous and chi-square or Fisher’s exact test in case of categorical variables. Abbreviations: CTSI, CT severity index; BUN, blood urea nitrogen; LDH, lactate dehydrogenase; hsCRP, high-sensitivity C-reactive protein; IL-6, interleukin 6; RDW, red blood cell distribution width; Ly/neu, lymphocyte/neutrophil; AA, amino acid; FE, fractional excretion.

**Table 4:** Predictors of mechanical ventilation.

Parameter	B	*p*
**Models 1 and 2**		
CTSI	0.261	0.004
Plasma Gly	−0.071	0.028
FE Gly	−1.200	0.053
FE Arg	0.141	0.101
**Model 3**		
CTSI	3.496	0.090
Plasma glucose	−3.635	0.148
Troponin T	0.218	0.128
IL-6	−0.023	0.073
Ly neu	40.809	0.123
Plasma Ser	−0.682	0.143
Plasma Gly	−0.405	0.108
Plasma Phe	0.156	0.111
FE Gly	−10.758	0.090
FE Arg	0.917	0.124
LDH	−0.003	0.178
Serum creatinine	−0.210	0.108
**Model 4**		
CTSI	0.856	0.005
Plasma glucose	−0.470	0.067
BUN	−0.243	0.053
Troponin T	0.049	0.038
IL-6	−0.006	0.090
Plasma Gly	−0.160	0.027
Plasma Phe	0.055	0.086
FE Gly	−2.390	0.019
LDH	−0.002	0.123

Logistic regression using backward LR method, B denotes the regression coefficient, while *p* denotes the statistical significance of the individual predictors. Model 1: serum creatinine, CTSI, plasma glucose, troponin T, hsCRP, IL-6, Ly/neu, plasma Ser, plasma Gly, plasma Phe, FE Gly, FE Arg. Model 2: BUN, CTSI, plasma glucose, troponin T, hsCRP, IL-6, Ly/neu, plasma Ser, plasma Gly, plasma Phe, FE Gly, FE Arg. Model 3: serum creatinine, CTSI, plasma glucose, troponin T, LDH, hsCRP, IL-6, Ly/neu, plasma Ser, plasma Gly, plasma Phe, FE Gly, FE Arg. Model 4: BUN, CTSI, plasma glucose, troponin T, LDH, hsCRP, IL-6, Ly/neu, plasma Ser, plasma Gly, plasma Phe, FE Gly, FE Arg. Abbreviations: CTSI, CT severity index; BUN, blood urea nitrogen; LDH, lactate dehydrogenase; hsCRP, high-sensitivity C-reactive protein; IL-6, interleukin 6; Ly/neu, lymphocyte/neutrophil ratio; FE, fractional excretion.

## Data Availability

Data is contained within the article.

## Supplementary Materials

The following are available online at https://www.mdpi.com/article/10.3390/metabo12060486/s1, Supplementary Figure S1. An Excel spreadsheet-based calculator for prediction of mortality or need of mechanical ventilation; Supplementary Table S1. Additional characteristics of study population by survival state; Supplementary Table S2. ROC analysis for survival state; Supplementary Table S3. Additional characteristics of study population by mechanical ventilation state; Supplementary Table S4. ROC analysis for mechanical ventilation state.
