# Supplementary material for: Plasma Levels and Renal Handling of Amino Acids Contribute to Determination of Risk of Mortality or Feed of Ventilation in Patients with COVID-19
Source: Metabolites. 2022 May 27;12(6):486. doi: 10.3390/metabo12060486 (PMC9228241; doi:10.3390/metabo12060486)
Supplement: Supplementary file 1 [file metabolites-12-00486-s001.zip › metabolites-1698508 Supplementary Figure.pdf]

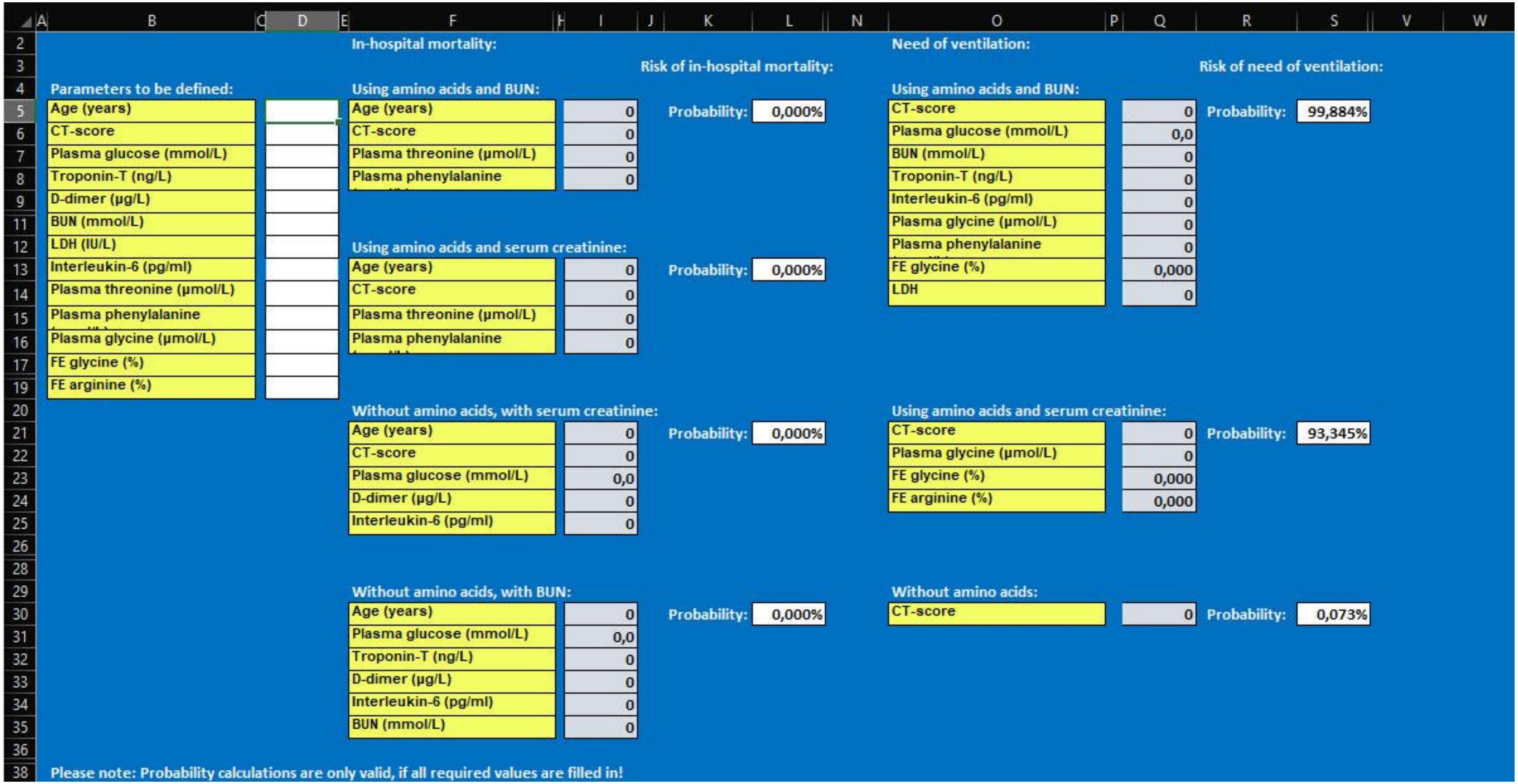

Supplementary Figure S1. An Excel spreadsheet-based calculator for prediction of mortality or need of mechanical ventilation.
