# Supplementary material for: Plasma Levels and Renal Handling of Amino Acids Contribute to Determination of Risk of Mortality or Feed of Ventilation in Patients with COVID-19
Source: Metabolites. 2022 May 27;12(6):486. doi: 10.3390/metabo12060486 (PMC9228241; doi:10.3390/metabo12060486)
Supplement: Supplementary file 1 [file metabolites-12-00486-s001.zip › metabolites-1698508 Supplementary Tables.pdf]

Supplementary Table S1. Additional characteristics of study population by survival state.

Parameter	Unit	Survivors<br>Median (q1–q3)	Non-survivors<br>Median (q1–q3)	p
Gender	male [%]	87 [53.4]	16 [61.5]	0.438
**Comorbidities**				
Kidney disease	n [%]	8 [4.8]	3 [11.5]	0.175
Autoimmune disease	n [%]	10 [6.1]	1 [3.8]	0.543
Na	mmol/l	137 (134-139)	136 (133-139)	0.405
K	mmol/l	4.16 (3.89-4.54)	4.63 (4.10-4.86)	0.012
eGFR	ml/min	85 (67-96)	63 (32-73)	<0.001
AST	U/l	33 (24-46)	42 (29-81)	0.034
ALT	U/l	28 (18-47)	27 (16-36.5)	0.347
LDH	U/l	534 (405-743)	677 (532-837)	0.015
Procalcitonin	ng/ml	0.06 (0.03-0.1)	0.17 (0.12-0.57)	<0.001
Ferritin	ug/l	462 (199-792)	564 (308-1345)	0.163
INR		1.08 (1.01-1.17)	1.15 (1.07-1.31)	0.014
WBC	G/l	5.74 (4.42-8.15)	8.73 (5.59-11.02)	0.006
Neutrophil	%	73.1 (63.3-81.0)	82.7 (74.9-87.9)	<0.001
Neutrophil abs	G/l	4.11 (2.94-6.41)	6.92 (4.28-9.50)	0.001
Lymphocyte	%	19.3 (13.1-27.9)	10.3 (6.9-16.5)	<0.001
Lymphocyte abs	G/l	1.14 (0.84-1.48)	0.78 (0.57-1.18)	0.006
Monocyte	%	5.8 (4.3-7.6)	4.7 (3.0-6.0)	0.018
Monocyte abs	G/l	0.34 (0.22-0.50)	0.37 (0.27-0.53)	0.253
Eosinophil	%	0.2 (0.0-1.2)	0.1 (0.0-0.7)	0.309
Eosinophil abs	G/l	0.01 (0.00-0.06)	0.01 (0.00-0.07)	0.544
Basophil	%	0.3 (0.2-0.4)	0.2 (0.1-0.3)	0.005
Basophil abs	G/l	0.02 (0.01-0.03)	0.01 (0.01-0.02)	0.472
RBC	T/l	4.82 (4.39-5.20)	4.62 (4.12-4.94)	0.098
Hb	g/l	142 (130-154)	135 (108-144)	0.020
Htc	%	41.4 (38-44.4)	40.1 (35.4-43.1)	0.056
MCV	fl	85.4 (83.0-88.4)	85.7 (81.9-89.5)	0.921
MCH	pg	29.5 (28.6-30.4)	29.0 (28.3-29.9)	0.157
MCHC	g/l	345 (337-351)	338 (327-349)	0.018
Thrombocyte	G/l	209 (163-276)	184 (138-323)	0.596
MPV	fl	10.4 (9.8-11.2)	10.6 (9.9-11.3)	0.575
Neu/mo		12.21 (8.50-18.37)	16.93 (13.10-20.23)	0.006
Plasma Asp	µmol/l	5.0 (4.0-6.3)	6.6 (5.0-9.0)	0.001
Plasma Glu	µmol/l	61.4 (46.1-81.0)	64.9 (48.8-100.0)	0.235
Plasma Asn	µmol/l	33.9 (29.2-37.8)	31.7 (27.5-38.0)	0.314
Plasma Gln	µmol/l	387.32 (331-431)	362.1 (298.0-407.0)	0.171
Plasma Hys	µmol/l	46.7 (38.4-53.9)	42.9 (35.9-49.0)	0.096
Plasma Gly	µmol/l	118.2 (98.0-142.1)	98.8 (83.8-112.0)	0.001
Plasma Arg	µmol/l	18.0 (12.5-23.7)	16.3 (13.0-21.0)	0.409
Plasma Ala	µmol/l	258.0 (223.6-298.0)	267.7 (226.0-318.0)	0.858
Plasma Tyr	µmol/l	50.0 (42.7-58.4)	56.1 (46.3-68.0)	0.029
Plasma Cys	µmol/l	30.1 (18.7-41.0)	33.3 (23.0-42.7)	0.180
Plasma Val	µmol/l	192.9 (166.2-223.0)	198.8 (180.2-225.0)	0.343
Plasma Met	µmol/l	22.7 (18.8-26.7)	22.9 (20.8-28.1)	0.396
Plasma Trp	µmol/l	51.3 (45.2-60.9)	44.8 (34.0-53.0)	0.005
Plasma Ile	µmol/l	59.4 (50.0-74.4)	65.0 (53.0-74.7)	0.363
Plasma Leu	µmol/l	123.0 (104.6-143.9)	129.1 (109.9-146.0)	0.365
Plasma Lys	µmol/l	85.2 (74.0-100.0)	79.9 (69.0-96.0)	0.180
Plasma Pro	µmol/l	167.61 (113.43-235)	192.0 (128.5-249.0)	0.453
Plasma total AA	µmol/l	1981.9 (1780.8-2146.8)	1897.3 (1757.9-2185.6)	0.838
Plasma Tyr/Phe	µmol/µmol	0.643 (0.539-0.766)	0.564 (0.479-0.624)	0.004
Urinary total AA/creatinine	µmol/mmol	0.250 (0.188-0.341)	0.189 (0.137-0.358)	0.063
FE Asp	%	5.577 (3.529-8.216)	6.369 (4.069-10.072)	0.420
FE Glu	%	0.477 (0.337-0.703)	0.531 (0.376-0.854)	0.220

FE Asn	%	2.354 (1.406-3.294)	3.401 (1.922-4.350)	0.020
FE Ser	%	2.077 (1.541-2.866)	2.316 (1.665-3.491)	0.274
FE_Gln	%	0.542 (0.367-0.715)	0.447 (0.249-0.672)	0.159
FE Hys	%	4.492 (3.042-7.322)	3.789 (2.323-5.873)	0.321
FE Gly	%	2.548 (1.792-3.896)	1.999 (1.660-3.079)	0.104
FE Thr	%	0.955 (0.651-1.287)	1.151 (0.750-1.519)	0.216
FE Ala	%	0.432 (0.309-0.672)	0.527 (0.371-0.658)	0.368
FE Tyr	%	1.181 (0.783-1.590)	1.021 (0.756-1.682)	0.744
FE Cys	%	4.163 (2.614-6.292)	3.833 (3.108-6.530)	0.773
FE Val	%	0.37 (0.2615-0.499)	0.447 (0.254-0.727)	0.101
FE Met	%	1.647 (1.035-2.306)	2.335 (1.201-3.250)	0.021
FE Trp	%	2.284 (1.196-3.401)	2.786 (1.737-4.081)	0.135
FE Phe	%	0.985 (0.698-1.490)	1.366 (0.637-1.961)	0.173
FE Ile	%	0.462 (0.327-0.637)	0.625 (0.410-0.771)	0.032
FE Leu	%	0.331 (0.259-0.461)	0.475 (0.293-0.650)	0.009
FE Lys	%	1.227 (0.787-1.696)	1.770 (1.160-2.490)	0.006
FE Pro	%	0.046 (0.028-0.068)	0.089 (0.044-0-157)	0.002

p, survivors vs. non-survivors, using Mann-Whitney U test in case of continuous and chi-square or Fisher's exact test in case of categorical variables. Abbreviations: DM, diabetes mellitus; CV, cardiovascular; eGFR, estimated glomerular filtration rate; AST, aspartate transaminase; ALT, alanine transaminase; LDH, lactate dehydrogenase; INR, international normalized ratio; WBC, white blood cell; RBC, red blood cell; Hb, hemoglobin; Htc, hematocrite; MCV, mean corpuscular volume; MCH, mean corpuscular hemoglobin; MCHC, mean corpuscular hemoglobin concentration; MPV, mean platelet volume; Neu/mo, neutrophil/monocyte; AA, amino acid; FE, fractional excretion.

Supplementary Table S2. ROC analysis for survival state.

Parameter	Area	p	Cut-off	Sensitivity	Specificity
				%	%
Age	0.795	<0.001	72	65.4	86.3
CTSI	0.732	<0.001	13.5	59.1	77.9
K	0.654	0.012	4.61	53.8	79.3
Plasma glucose	0.765	<0.001	7.6	73.9	74.4
BUN	0.829	<0.001	5.32	92.3	66.0
Serum creatinine	0.663	0.008	92	61.5	73.3
eGFR	0.759	<0.001	84	53.1	92.3
AST	0.637	0.034	59	43.5	85.0
Troponin T	0.850	<0.001	11.18	90.9	72.0
D-dimer	0.778	<0.001	751	91.3	53.7
LDH	0.657	0.015	600.5	69.6	60.3
hsCRP	0.740	<0.001	42.4	88.0	53.4
Procalcitonin	0.793	<0.001	0.105	83.3	76.7
IL-6	0.825	<0.001	49.15	78.3	75.5
INR	0.656	0.004	1.045	95.8	38.0
WBC	0.667	0.006	8.26	57.7	76.4
Neutrophil	0.720	<0.001	80.65	65.4	72.7
Neutrophil abs	0.700	0.001	6.06	61.5	73.3
Lymphocyte	0.738	<0.001	11.45	82.4	57.7
Lymphocyte abs	0.669	0.006	0.785	79.4	53.8
Monocyte	0.645	0.018	6.35	41.2	88.5
Basophil	0.667	0.005	0.25	55.2	69.2
Hb	0.642	0.020	141.5	51.5	73.1
MCHC	0.644	0.032	331.5	90.9	38.5
RDW	0.772	<0.001	14.35	61.5	90.3
Ly/neu	0.739	<0.001	0.138	81.2	57.7
Neu/mo	0.669	0.006	12.7	88.5	55.8
Plasma Asp	0.694	0.002	5.76	65.4	65.5
Plasma Ser	0.724	<0.001	63.04	63.6	73.1
Plasma Gly	0.696	0.001	106.1	64.2	73.1
Plasma Thr	0.738	<0.001	64.2	77.6	65.4
Plasma Trp	0.671	0.005	39.1	89.7	42.3
Plasma Tyr	0.633	0.029	52.7	65.4	63.0
Plasma Phe	0.785	<0.001	78.7	96.2	52.1
Plasma Tyr/Phe	0.676	0.004	52.7	65.4	63.0
FE Asn	0.642	0.020	3.84	42.3	86.8
FE Arg	0.718	<0.001	4.27	69.2	67.3
FE Met	0.642	0.021	2.74	46.2	84.9
FE Ile	0.631	0.032	0.60	61.5	69.2
FE Leu	0.661	0.009	0.42	65.4	71.7
FE Lys	0.667	0.006	1.46	65.4	64.8
FE Pro	0.691	0.002	1.46	65.4	64.8

Abbreviations: CTSI, CT severity index; BUN, blood urea nitrogen; eGFR, estimated glomerular filtration rate; AST, aspartate transaminase; LDH, lactate dehydrogenase; hsCRP, high-sensitivity C-reactive protein; IL-6, interleukin 6; INR, international normalized ratio; WBC, white blood cell; Hb, hemoglobin; MCHC, mean corpuscular hemoglobin concentration; RDW, reticulocyte distribution width; Ly/neu, lymphocyte/neutrophil; Neu/mo, neutrophil/monocyte; FE, fractional excretion.

Supplementary Table S3. Additional characteristics of study population by mechanical ventilation state.

		No mechanical ventilation	Mechanical ventilation	
**Parameter**	**Unit**	**Median (q1–q3)**	**Median (q1–q3)**	**p**
Age	year	60.2 (46.2-69.95)	65.17 (51.78-73.41)	0.182
Gender	male [%]	93 [53.4]	10 [66.7]	0.324
**Comorbidities**				
CV disease	n [%]	33 [18.8]	5 [33.3]	0.153
Kidney disease	n [%]	10 [5.7]	1 [6.7]	0.604
Malignancy	n [%]	12 [6.8]	1 [6.7]	0.729
Autoimmune disease	n [%]	10 [5.7]	1 [6.7]	0.604
Na	mmol/l	137 (134-139)	135 (133-138)	0.134
K	mmol/l	4.17 (3.905-4.565)	4.68 (3.92-5.05)	0.073
eGFR	ml/min	84 (66-95)	64 (43-71)	0.003
AST	U/l	33 (24-46)	63 (42-93)	<0.001
ALT	U/l	27 (18-46)	31 (26-46)	0.486
D-dimer	µg/l	732 (427-1202)	1306 (784-1957)	0.013
Ferritin	ug/l	459 (199-774)	702 (379-1345)	0.050
INR		1.08 (1.02-1.17)	1.17 (1.08-1.25)	0.032
WBC	G/l	5.76 (4.36-8.31)	10.32 (6.47-11.80)	0.005
Neutrophil	%	74.0 (64.2-81.6)	84.0 (76.5-88.4)	0.006
Neutrophil abs	G/l	4.18 (2.94-6.545)	6.81 (5.15-9.66)	0.004
Lymphocyte	%	18.6 (12.3-27.0)	9.6 (7.1-18.4)	0.010
Lymphocyte abs	G/l	1.13 (0.80-1.48)	0.98 (0.74-1.13)	0.113
Monocyte	%	5.8 (4.3-7.6)	3.8 (2.8-5.5)	0.004
Monocyte abs	G/l	0.36 (0.22-0.51)	0.32 (0.28-0.48)	0.813
Eosinophil	%	0.2 (0.0-1.15)	0.1 (0.0-0.3)	0.172
Eosinophil abs	G/l	0.01 (0.00-0.06)	0.01 (0.00-0.025)	0.255
Basophil	%	0.3 (0.2-0.4)	0.2 (0.1-0.3)	0.006
Basophil abs	G/l	0.02 (0.01-0.03)	0.01 (0.01-0.02)	0.324
RBC	T/l	4.82 (4.38-5.19)	4.66 (4.16-5.16)	0.473
Hb	g/l	141 (130-154)	139 (130-145)	0.280
Htc	%	41.0 (37.8-44.3)	40.3 (37.4-43.1)	0.432
MCV	fl	85.4 (82.9-88.6)	86.2 (82.6-89.2)	0.886
MCH	pg	29.5 (28.6-30.4)	28.7 (28.1-29.8)	0.189
MCHC	g/l	345 (337-351)	336 (330-347)	0.082
Thrombocyte	G/l	210 (159-281)	174 (149-257)	0.259
MPV	fl	10.4 (9.8-11.2)	11.0 (10.1-12.2)	0.066
Neu/mo		12.37 (8.57-18.33)	17.46 (13.22-30.68)	0.007
Plasma Glu	µmol/l	61.4 (45.5-81.0)	85.0 (62.5-101.2)	0.013
Plasma Asn	µmol/l	33.68 (29.2-37.9)	30.0 (26.6-37.5)	0.162
Plasma Gln	µmol/l	388.2 (333.0-435.4)	328.0 (283.9-367.5)	0.004
Plasma Hys	µmol/l	46.7 (38.4-53.8)	38.0 (34.0-45.2)	0.010
Plasma Thr	µmol/l	74.4 (63.8-90.8)	60.0 (49.9-74.0)	0.005
Plasma Arg	µmol/l	18.0 (12.5-23.7)	13.5 (12.8-18.6)	0.140
Plasma Ala	µmol/l	258.1 (222.9-298.0)	273.2 (227.3-312.5)	0.741
Plasma Tyr	µmol/l	50.8 (43.0-58.5)	53.0 (45.7-67.5)	0.253
Plasma Cys	µmol/l	30.9 (18.8-41.0)	31.0 (23.0-44.4)	0.276
Plasma Val	µmol/l	193.1 (166.2-223.5)	186.0 (177.6-215.9)	0.921
Plasma Met	µmol/l	22.7 (19.0-26.6)	24.0 (18.5-28.6)	0.653
Plasma Trp	µmol/l	51.3 (44.7-60.9)	44.6 (35.5-51.5)	0.007
Plasma Ile	µmol/l	59.9 (50.4-74.5)	61.0 (49.0-74.5)	0.944
Plasma Leu	µmol/l	123.9 (104.7-144.0)	125.0 (106.0-145.8)	0.819
Plasma Lys	µmol/l	85.7 (74.0-100.0)	78.0 (68.0-84.0)	0.149
Plasma Pro	µmol/l	169.1 (117.6-239.6)	182.4 (145.5-221.5)	0.706
Plasma total AA	µmol/l	1992.2 (1779.2-2175.8)	1859.0 (1757.5-2020.4)	0.261
Plasma Tyr/Phe	µmol/µmol	0.639 (0.524-0.761)	0.581 (0.504-0.611)	0.018
FE Asp	%	5.580 (3.601-8.221)	6.567 (3.942-9.459)	0.658
FE Glu	%	0.482 (0.341-0.723)	0.444 (0.360-0.723)	0.763
FE Asn	%	2.364 (1.436-3.478)	3.317 (1.904-4.108)	0.298

FE Ser	%	2.207 (1.571-2.958)	1.686 (1.278-2.512)	0.200
FE Hys	%	4.550 (3.024-7.348)	3.223 (2.099-4.244)	0.035
FE Thr	%	0.972 (0.679-1.333)	0.750 (0.618-1.205)	0.259
FE Ala	%	0.448 (0.310-0.675)	0.418 (0.320-0.562)	0.558
FE Tyr	%	1.185 (0.790-1.600)	0.901 (0.744-1.215)	0.190
FE Cys	%	4.129 (2.586-6.326)	3.842 (3.468-6.382)	0.421
FE Val	%	0.376 (0.261-0.513)	0.421 (0.302-0.590)	0.475
FE Met	%	1.663 (1.027-2.391)	2.208 (1.373-3.828)	0.040
FE Trp	%	2.298 (1.210-3.410)	3.189 (1.683-4.859)	0.156
FE Phe	%	0.987 (0.674-1.529)	1.237 (0.835-1.718)	0.338
FE Ile	%	0.474 (0.326-0.657)	0.625 (0.467-0.748)	0.070
FE Leu	%	0.334 (0.259-0.480)	0.461 (0.368-0.592)	0.017
FE Lys	%	1.266 (0.789-1.793)	1.728 (1.342-2.169)	0.034
FE Pro	%	0.046 (0.028-0.070)	0.084 (0.054-0.121)	0.008

p, no mechanical ventilation vs. mechanical ventilation, using Mann-Whitney U test in case of continuous and chi-square or Fisher's exact test in case of categorical variables. Abbreviations: DM, diabetes mellitus; CV, cardiovascular; CTSI, CT severity index; BUN, blood urea nitrogen; eGFR, estimated glomerular filtration rate; AST, aspartate transaminase; ALT, alanine transaminase; LDH, lactate dehydrogenase; hsCRP, high-sensitivity C-reactive protein; IL-6, interleukin 6; INR, international normalized ratio; WBC, white blood cell; RBC, red blood cell; Hb, hemoglobin; Htc, hematocrite; MCV, mean corpuscular volume; MCH, mean corpuscular hemoglobin; MCHC, mean corpuscular hemoglobin concentration; RDW, reticulocyte distribution width; MPV, mean platelet volume; Ly/neu, lymphocyte/neutrophil; Neu/mo, neutrophil/monocyte; AA, amino acid; FE, fractional excretion.

Supplementary Table S4. ROC analysis for mechanical ventilation state.

Parameter	Area	p	Cut-off	Sensitivity	Specificity
				%	%
CTSI	0.920	<0.001	11.5	100	67.4
Plasma glucose	0.796	<0.001	7.01	84.6	65.3
BUN	0.776	<0.001	5.4	86.7	63.6
Serum creatinine	0.725	0.004	82.5	86.7	56.4
eGFR	0.732	0.003	73.5	65.9	87.6
AST	0.775	0.001	59	61.5	84.7
Troponin T	0.851	<0.001	9.67	100	62.7
D-dimer	0.699	0.013	648	92.9	45.0
LDH	0.773	0.001	770	71.4	77.6
hsCRP	0.808	<0.001	42	100	52.0
Procalcitonin	0.844	<0.001	0.11	93.3	74.9
Ferritin	0.663	0.050	217	100	27.9
IL-6	0.836	<0.001	70.3	78.6	79.6
INR	0.672	0.002	1.045	100	36.3
WBC	0.720	0.005	10.1	53.3	85.2
Neutrophil	0.715	0.006	81.1	66.7	73.9
Neutrophil abs	0.727	0.004	5.95	73.3	71.6
Lymphocyte	0.750	0.010	14.4	65.9	78.6
Monocyte	0.725	0.004	4.65	69.9	66.7
Basophil	0.712	0.006	0.15	83.0	46.7
RDW	0.749	0.001	13.95	73.3	82.4
Ly/neu	0.757	0.001	0.18	66.5	78.6
Neu/mo	0.712	0.007	12.5	93.3	51.1
Plasma Asp	0.815	0.007	6.88	80.0	79.0
Plasma Glu	0.694	0.013	66.8	73.3	62.5
Plasma Ser	0.784	<0.001	67.0	49.4	100
Plasma Gln	0.724	0.004	378	55.1	93.3
Plasma Hys	0.702	0.010	41.0	68.8	66.7
Plasma Gly	0.803	<0.001	106	63.1	86.7
Plasma Thr	0.718	0.005	60.0	81.8	53.3
Plasma Trp	0.712	0.007	46.2	69.9	66.7
Plasma Phe	0.778	<0.001	79.1	100	51.1
Plasma Tyr/Phe	0.684	0.018	0.655	47.2	100
Urinary total AA/creatinine	0.758	<0.001	0.233	58.2	93.3
FE Gln	0.741	<0.001	0.53	54.7	86.7
FE Hys	0.662	0.037	3.81	58.0	73.3
FE Gly	0.740	0.002	2.03	69.2	80.0
FE Arg	0.733	0.003	5.75	60.0	81.1
FE Met	0.662	0.038	2.98	46.7	86.4
FE Leu	0.690	0.015	0.43	73.3	70.4
FE Lys	0.670	0.030	1.30	80.0	53.8
FE Pro	0.713	0.006	0.05	86.7	56.8

Abbreviations: CTSI, CT severity index; BUN, blood urea nitrogen; eGFR, estimated glomerular filtration rate; AST, aspartate transaminase; LDH, lactate dehydrogenase; hsCRP, high-sensitivity C-reactive protein; IL-6, interleukin 6; WBC, white blood cell; RDW, reticulocyte distribution width; Ly/neu, lymphocyte/neutrophil; Neu/mo, neutrophil/monocyte; AA, amino acid; FE, fractional excretion.
